# Hydrometrocolpos and Post-axial Polydactyly Complicated With Acute Intestinal Obstruction and Hydroureteronephrosis

**DOI:** 10.7759/cureus.17612

**Published:** 2021-08-31

**Authors:** Liliana Arriola-Montenegro, Jose Arriola-Montenegro, Maria Pia-Balmaceda, Carlos Celis-Albujar, Adrian Riva-Moscoso, Patricia Cabanillas-Lozada, Vladimir Velásquez-Huarcaya

**Affiliations:** 1 Internal Medicine, Universidad Peruana de Ciencias Aplicadas, Lima, PER; 2 Internal Medicine, Sociedad Nacional de Capacitacion, Lima, PER; 3 Internal Medicine, Instituto de Investigación Nutricional, Lima, PER; 4 Surgery, Escuela de Medicina, Universidad Peruana de Ciencias Aplicadas (UPC), Lima, PER; 5 Pediatrics, Universidad Nacional de Trujillo, Lima, PER; 6 Pediatrics, Universidad Privada Antenor Orrego, Lima, PER

**Keywords:** case reports, hydrometrocolpos, infant, mckusick kaufman syndrome, polydactyly

## Abstract

This report presents a case of a one-month three-day-old full-term female infant with hydrometrocolpos (HMC) and post-axial polydactyly whose first clinical sign was acute intestinal obstruction and hydroureteronephrosis, caused by compression of the structures due to the increasing size of the cystic-like pelvic mass. This is the first report of HMC with post-axial polydactyly complicated with acute intestinal obstruction in Peru. It raises importance on prenatal diagnosis, management and complications of HMC. Although it is rare, clinicians should have it as an option when discussing abdominal cystic masses in neonates to perform early management and avoid complications. Continuous follow-up should be carried out on patients presenting with HMC and post-axial polydactyly to assess for Bardet-Biedl syndrome, which could affect different systems in those patients long-term.

## Introduction

Hydrometrocolpos (HMC) is an uncommon pathology worldwide that consists of the accumulation of fluid in the uterus and vagina due to some malformation that blocks the drainage of cervical secretions. HMC, when is accompanied by post-axial polydactyly, may be related to certain syndromes such as McKusick-Kaufman syndrome (MKKS), which more often presents in children between 5 and 10 years old. The difference between them varies according to other clinical presentations. [1]. MKKS presents with HMC, polydactyly and cardiac malformations [2] while hypogonadism, obesity, cone and rod dystrophy, renal dysfunction and cognitive impairment are also found in Bardet-Biedl syndrome (BBS) [3]. Mechanical compression of structures due to the enlarged uterus may cause hydronephrosis and even intestinal obstruction in certain cases [4]. This is the first known case of an infant with HMC and post-axial polydactyly complicated with acute intestinal obstruction and hydroureteronephrosis reported in Peru.

## Case presentation

A one-month and three-day-old full-term female infant was admitted to the emergency room due to five days of progressive abdominal distension. One day before being admitted to the hospital, she presented alimentary vomiting and obstipation. Physical examination revealed marked abdominal distension with collateral circulation and a palpable mass in the lower hemiabdomen of soft consistency with regular margins of 8 cm approximately.

**Figure 1:**
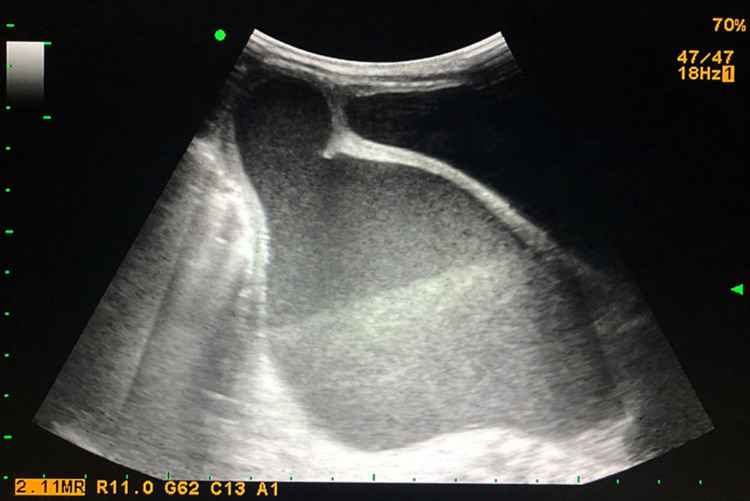
Patient's ultrasound with evidence of hydro-hematocolpos that extended into the vaginal canal

**Figure 2:**
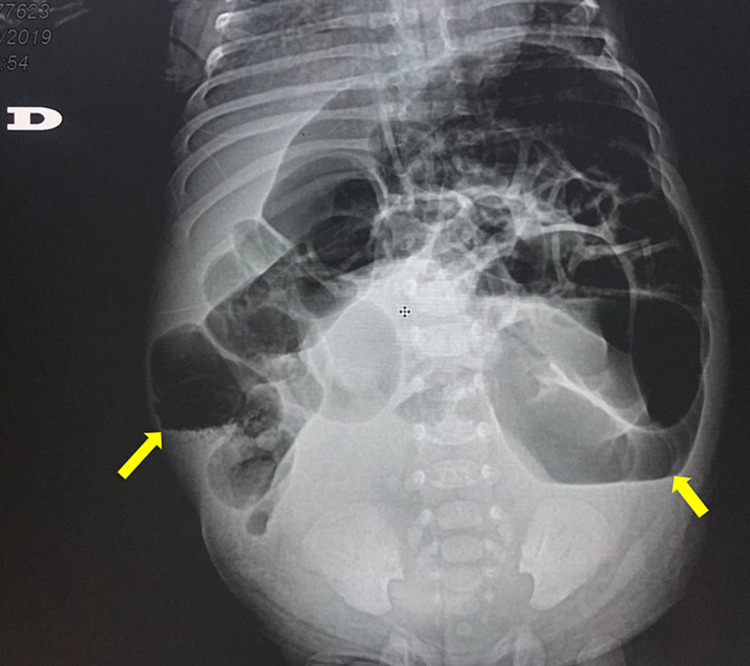
Patient's abdominal CT scan with evidence of small bowel distension

In addition, the urethral orifice is evidenced in the inferior middle third of the vulvar vestibule and vaginal introitus is not distinguished. Also, post-axial polydactyly is evidenced in all extremities. After admission, an abdominal ultrasound (Figure 1) and computed tomography (Figure 2) were performed.

 

Findings suggested hydro-hematocolpos which extended into the vaginal canal with no distinguishable communication or fistula between the bladder and vagina. Additionally, a congenital bilateral grade IV hydroureteronephrosis was observed. Pediatric surgery was consulted, who performed a colpostomy in which clear fluid is drained from the uterine cavity; drainage was placed. Two weeks later, a brownish foul-smelling draining material was evident. Immediately, an emergency exploratory laparotomy was performed, in which purulent material was obtained after performing a hysterotomy. The diagnosis of infected HMC was confirmed. Consequently, a permanent drain was placed, which requires monthly replacement. One week after surgery, a pelvic MRI was performed with suggestive findings of hydro-hematometra, which compresses the rectum, bowel loops and ovaries. The patient is still to be programmed for definitive surgery by specialists.

## Discussion

HMC is a malformation caused by vaginal atresia, imperforate hymen or a transverse vaginal membrane [5]. Although there is literature reporting HMC as a single malformation [6], most of them are associated with post-axial polydactyly [7] consisting of extra fingers and toes in the ulnar and fibular region, respectively. Some syndromes present both HMC and post-axial polydactyly such as MKKS [8], BBS, Ellis-van Creveld (EVC) or Pallister-Hall (PHS), where MKKS is the most common with an approximate incidence of 1:10,000 [2]. MKKS presents with the triad of HMC, post-axial polydactyly and cardiac malformations. Similarly, BBS presents cone and rod dystrophy in up to 90% to 100% of cases, hypogonadism, obesity - which appears at two to three years of age, renal dysfunction and decreased cognitive impairment as an additional finding to the primary triad [3]. These manifestations usually appear between 5 and 10-year-old patients and their prevalence is around 1:140,000 in North Americans and European populations with an increase in a consanguineous population [3]. Hence, a diagnosis of MKKS cannot be made until five years of age, probably due to the overlapping of symptoms with the other syndromes mentioned before [1].

Antenatal diagnosis of HMC is usually challenging as it is ultrasound dependent. Usually made during gynecological visits since the third trimester of pregnancy and can be misdiagnosed with other abdominal fetal cysts [9]. Similarly, post-axial polydactyly and congenital cardiac malformations can also be identified with antenatal ultrasounds. However, imaging is not considered the gold standard to confirm the diagnosis of MKKS, as these findings are considered only suggestive. Furthermore, patients should be followed up for at least five years with periodic ophthalmologic and renal evaluations to elaborate a differential diagnosis of BBS as mentioned above [2]. Newborns with HMC could be asymptomatic at birth, which can make the diagnosis difficult if findings are not obvious during prenatal ultrasounds. This disorder could be diagnosed in some patients as diverse complications arise [10] like this particular case, which happens due to continued estrogenic stimulation by the mother, more cervical and uterine secretions accumulate and could cause compression of certain adjacent structures within the infant. Cases of hydronephrosis [11], intestinal obstruction [4], rupture and infection of the HMC [5] have been reported as in this patient, which complicated the course of management. 

Management should be performed with early drainage of the HMC, either by hymenectomy or laparotomy for patients with a high vaginal obstruction or resolution of associated abdominal complications. A quick and temporary decompression with an aspiration of the cystic mass or vaginostomy, which reduces the chances of sepsis, is a complication that has been reported before [5,9]. Previously reported cases of HMC presenting with upper abdominal obstruction were resolved with vaginostomy and drainage of the HMC reducing the intestinal compression [4]. Some literature recommends delaying surgery until the patient reaches menarche if they are asymptomatic since the anatomical obstruction could be easily recognized in older patients [12]. This patient had a complication due to an over-infected HMC that led to sepsis in the infant. A laparotomy was performed to drain the HMC and clean the affected area. Although this case presents with HMC and post-axial polydactyly, it is too early to determine whether these findings are isolated or belong to any of the syndromes mentioned above. As recommended, a second surgery correcting the specific malformation should be performed [12].

## Conclusions

This is the first report of HMC with post-axial polydactyly complicated with hydroureteronephrosis in Peru, where its prevalence is unknown. This case highlights the importance of prenatal diagnosis, management and complications of HMC. Although it is a rare disease, clinicians should consider this pathology as a differential diagnosis when discussing abdominal cystic masses in neonates in order to perform early management and avoid complications, as contemplated in this case. We further recommend the periodic follow-up on patients presenting with HMC and post-axial polydactyly as it could be part of the BBS syndrome which could affect different systems in those patients long-term. The association of HMC and post-axial polydactyly represents a challenging diagnosis. Although clinical manifestations can be observed at birth, a definitive diagnosis can only be made several years later. Surgical management remains the mainstay of treatment.
